# The links between health-related behaviors and life satisfaction in elderly individuals who prefer institutional living

**DOI:** 10.1186/1472-6963-7-30

**Published:** 2007-02-27

**Authors:** Serap Inal, Feryal Subasi, Serap M Ay, Osman Hayran

**Affiliations:** 1 School of Physical Education and Sports, İstanbul University, 34310 Avcılar, Istanbul, Turkey; 2 Department of Health Education, Faculty of Health Education, Marmara University, 34865, Kartal, Istanbul, Turkey; 3 School of Physical Education and Sports, Marmara University, Anadolu Hisarı, Beykoz, Istanbul, Turkey; 4 Department of Public Health, School of Medicine, Marmara University, 34668, Haydarpasa, Istanbul, Turkey

## Abstract

**Background:**

Life satisfaction among residents of institutions is becoming an important issue in a rapidly aging population. The aim of this cross-sectional study was to investigate the links between life satisfaction and health-related behaviors amongst functionally independent elderly people who prefer institutional living in İstanbul, Turkey.

**Methods:**

The socio-demographic characteristics, health-related behaviors, leisure-time activities and fall histories of 133 residents of an institution in Istanbul were assessed by a structured questionnaire during face-to-face interviews. A validated life-satisfaction index questionnaire (LSI-A) was completed.

**Results:**

The mean age of the study group was 73.9 ± 8.0 (range 60–90 years). Within the group, 22.6% had never married and 14.3% had university degrees. The majority (71.4%) were in the low income bracket. The overall mean LSI-A score was 20.3 ± 5.9. Participants who declared moderate/high income levels had a significantly higher mean LSI-A score than those in the low-income bracket (p = 0.009). Multivariate analysis of the data suggested that leisure-time activities and participation in regular physical activities are significant predictors of LSI-A scores (R^2^: 0.112; p = 0.005 and p = 0.02, respectively).

**Conclusion:**

The findings imply that regular physical activity and leisure-time activities are significantly related to life satisfaction among residents in institutions. Participation in physical activity and leisure-time activity programs may help to improve the life satisfaction of elderly people living in institutions.

## Background

One of the main features of the world population in the late 20^th ^and early 21^st ^centuries has been the considerable increase in absolute and relative numbers of the elderly in both developed and developing countries. According to the World Health Organization, the number of elderly worldwide in 2004 was approximately 580 million and this figure is expected to increase in the coming years [1]. The percentage of people over the age of 65 in Turkey in 1990 was 4.3%, and this figure rose to 5.8% in 2003 and is estimated to represent 9.8% of the total population by 2025 [2,3]. Turkey tends to be a traditional culture with a collectivist orientation. The social structure is based on close-knit family relationships. Children and other relatives are expected to provide for the needs of older adults [4]. Therefore, only a small minority (approximately 4%) of the elderly population prefer institutional care [5]. However, the demand for institutional care has been increasing owing to the economic and medical problems of the older population, changing family lifestyles and the scarcity of available institutional services. It is notable that those who prefer institutional care are usually widowed, divorced or never married, have no children or close relatives, and are in the low-income bracket [3,6,7]. Nearly three out of every five elderly people living in institutions have low income status [5].

As an overview, the total bed capacity of institutions in the country that accept people aged 60 or over is approximately eighteen thousand, and such institutions can currently be classified into two groups: the first can be termed 'Elderly Care and Rehabilitation Centers', established for older people functionally dependent in respect of Activities of Daily Living (ADL) (bathing, dressing, continence, feeding, ambulation, etc); the second can be termed 'Nursing Homes', established for the elderly who are functionally independent or partially independent but need some assistance and supervision in ADL. Psycho-social services, basic health services, leisure time activities etc. are provided to the residents who are admitted to the institutions by nurses, social workers, psychologists and handicraft and sports instructors [5,8].

It is obvious that the social and medical problems of the elderly, whose population is increasing rapidly in Turkey, will become an important issue in the near future. In addition to longevity itself, quality of life is also important; this entails fulfillment of personal values and enjoyment of leisure time, and is influenced by physical, psychological, mental, social and economic circumstances [9,10]. As Ebersole [11] noted, the quality of life that leads to life satisfaction has become a reliable tool for investigating the efficiency of health care services and the effectiveness of rehabilitation programs.

The term 'quality of life' refers to an evaluation of the life conditions of a person, group or population. Objective or normative criteria can be used to measure quality of life, and these usually involve the quality of the physical and social environment, physical and mental health, and available support systems. However, subjective criteria, such as how the person considers his/her life, can also be used [12]. Subjective quality of life can be defined in terms of life satisfaction (LS), subjective well-being and happiness, etc. [12-14]. Life satisfaction, which includes factors such as health, education, interpersonal relationships and socio-economic status, is believed to be an evaluation of life in general [14].

Various studies have shown that limited ability to perform ADL also means decreased life satisfaction. For instance, among the elderly population in Sweden, those with reduced ADL capacity report several diseases and functional impairments that can cause low life-satisfaction [14]. Sato et al. [15] observed greater life satisfaction in Japanese pensioners living at home who had high levels of ADL.

There is a significant relationship between physical activity, functional status and health status in the elderly [16-18]. Decreases in these features are closely related to falls, which are a major problem area [19-23]. In addition, those who have had falls face the fear of falling again and tend to restrict their daily activities [22,24]. However, Laughton et al. [25] have reported that balance performance is not always a risk factor for elderly fallers; other factors should be considered, such as the risk levels in activities undertaken.

It has been reported that inactivity is also associated with greater behavioral risks and unhealthy lifestyles such as poor diet and smoking [26-28]. Tatum and colleagues [29] suggested detailed investigations into such health risks – unhealthy diet, excessive alcohol and tobacco consumption, lack of regular physical exercise, etc. – for promoting health in the elderly and for a significant change in their lifestyle in the nursing home. Health promotion in older people is a relatively new concept in geriatric care, and its importance has increased because of studies reporting that health-related behaviors could decrease the risk of secondary disabilities [30,31].

As far as we know, there are no data in the literature regarding life satisfaction status and health related behaviors, or the association between these two, in elderly residents of nursing homes in Turkey. There seems to be a need for additional investigation of the links between the health-related risks and life satisfaction among these residents. Thus, the aim of this cross-sectional study was to investigate the association between life satisfaction and health activities amongst functionally independent residents of nursing homes.

## Methods

### Subjects

This cross-sectional study was conducted as part of 'The Active Aging Project – Istanbul', funded by the Istanbul Greater Municipality. One hundred and thirty-three residents of the Municipality's Darulaceze Institute (48 females and 85 males, mean age 73.9 ± 8.0 [range: 60–90 yrs]) took part in the study. Elderly citizens with functional disabilities and/or independent in ADL are accepted by the Institute, which is located in Istanbul, the largest city in the country with a population of 10,072,447, constituting 16.02% of the total population, and has the largest bedding capacity of all such institutions in the country, as well as the largest professional staff including physicians, physical therapists, social workers, psychologists, nurses and nursing assistants and non-professional staff. Darulaceze Institute can be defined as 'Elderly Care and Rehabilitation Centers' according to institutional care system for elderly.

The following were the inclusion criteria for our study:

1. a stable medical condition;

2. not being bedridden or in a wheelchair;

3. independence in performing daily living activities (independence in walking indoors/outdoors, climbing up stairs, doing self care activities including taking a bath, feeding, dressing/undressing, were evaluated by self-reporting),

4. sufficient mental capacity and cognitive function to learn and retain new information;

5. willingness to participate in the study.

Functionally independent subjects were selected in order to collect accurate information from the prepared questionnaire and life satisfaction index.

All the participants and institution staff were informed about the objectives and methodology of the study, and the study plan was approved by the Ethics Committee of Marmara University. The participants were evaluated by institution's physicians before the study started. Individuals who had histories of significant cardiovascular, pulmonary, metabolic and/or musculo-skeletal diseases and/or psychiatric disorders were excluded. Among the 145 residents, 133 were eligible for the study, the participation rate being 92%. There were no internal dropouts during the study.

Data were collected during face-to-face interviews. The socio-demographic characteristics, health-related behaviors, leisure-time activities and fall histories of the participants were evaluated using a structured questionnaire.

The socio-demographic characteristics of the subjects were coded as follows: gender (female = 1; male = 2); marital status (1 = never married, divorced, widow/widower; 2 = married); level of education (1 = no education, 2 = primary school, 3 = high school, 4 = college/university); social security (1 = yes, 2 = no); and number of children. Income status was classified according to the participants' self-assessment.

Physical activity (walking, callisthenic exercises) were assessed in terms of frequency (sessions per week) and duration (minutes per session). Participants undertaking physical activity at least 3 times in a week with at least 30 minutes per session were classified as physically active, while the rest were classified as physically inactive.

Smoking habits were classified as 'current', 'ex-', and 'non-smoker'. Alcohol use was classified into four categories; abstainers, infrequent drinkers, moderate drinkers (1 or 2 glasses a day) and excessive drinkers (more than 2 glasses a day).

Any participant who had had at least one fall during the past year was considered to have a 'positive' fall history.

The frequency of leisure time activities (handicrafts, reading, gardening etc.) was categorized as " always", "frequently", "sometimes", "rarely" or "never". Participants who took part in leisure-time activities "always" or "frequently" were recorded as having regular leisure-time activities.

Life satisfaction was assessed by the LSI-A satisfaction index, which was first prepared, validated and published by Neugarten et al. [32] and was adapted and translated into Turkish by Karatas in 1988 as a highly reliable and valid index for the Turkish elderly population [33,34]. The internal consistency reliability coefficient (Cronbach's alpha) was computed at 0.66 for LSI-A in our study group, which was slightly below the lowest acceptable level (< 0.70). The LSI-A comprised twenty items, of which twelve were positively worded. The answers "disagree", "don't know" and "agree" were assigned as 0, 1 and 2 points respectively. Eight items were negatively worded, with 0 assigned to the "agree" answer. The total score obtainable from the LSI-A ranged between 0 and 40 points.

The collected data were analyzed using the SPSS 11.5 software package. Parametric tests of significance such as analysis of variance (ANOVA) and unpaired t tests were used for group comparisons. Linear regression models were constructed to determine LSI-A predictors. The variables which were found to be significantly related to LSI-A by univariate analysis, income level, regular physical and leisure time activity, were included in a multivariate analysis. The critical value for significance in all analyses was 0.05 (p < 0.05).

## Results

### Sample

As presented in Table 1, the mean age of the participants (133) was 73.9 ± 8.0 years; 36.1% were female and 63.9% were male. Almost half (45.9%) of the study group had no relatives or close friends and the majority (59.5%) had spent most of their lives in a big city.

**Table 1:** Mean LSI-A scores of the study group and socio-demographic characteristics (n = 133)

**Socio-demographic characteristics**	**N (%)**	**LSI-A score Mean (SD)**	**p-value**
**Gender **^**1**^			t = 0.056
Female	48 (36.1)	20.35(5.72)	p = 0.956
Male	85(63.9)	20.29(6.12)	
**Education Level **^**2**^			F = 0.69
No education/illiterate	37(27.8)	19.24(6.02)	P = 0.557
Primary school	62(46.6)	21.06(5.85)	
Secondary school	15(11.3)	20.00(6.04)	
University	19(14.3)	20.36(6.23)	
**Marital status**^**2**^			F = 1.16
Married	5(3.8)	21.60(4.33)	P = 0.325
Never married	30(22.6)	20.13(5.18)	
Divorced	33(24.8)	18.78(6.03)	
Widow/Widower	65(48.8)	21.07(6.30)	
**Social security**^**2**^			F = 1.41
Government retirement fund	13(9.8)	22.84 (6.98)	P = 0.234
Bag-Kur (Public)	6(4.5)	23.33(3.50)	
Workers' social insurance (Public)	31(23.3)	20.64(5.72)	
Green card (public insurance for the poor)	56(42.1)	19.83(6.10)	
No insurance	27(20.3)	19.03(5.57)	
**Income status (as self-assessed)**^**2**^			**t = -2.66**
Low	95(71.4)	19.46(5.73)	**P = 0.009**
Moderate/Good	38(28.6)	22.44(6.03)	

^1^Unpaired t test

^2 ^ANOVA (one-way)

### Life satisfaction

The mean LSI-A score for the whole group was 20.9 ± 5.7. A considerable percentage of the group (27.8%) was illiterate; 46.6% had received primary school education; 22.6% had never been married, 20.3% were without health insurance and 71.4% were in the low-income bracket. The participants with low income had a significantly lower mean LSI-A score than those with moderate/high income (p = 0.009). However, no statistically significant differences were observed between the mean LSI-A scores of the participants in respect of other socio-demographic characteristics (Table 1).

It was observed that 27.1% of the study group took part in regular physical activities (walking, calisthenics) and 25.6% took part in regular leisure-time activities (handicrafts, reading, and gardening). Only 21.8% among the elderly had had a fall in the previous year. 35.3% were non-smokers and 78.2% were teetotal. The elderly who performed regular physical activity had significantly higher mean LSI-A scores than those who did not (p = 0.01). Those participating in regular leisure-time activities also had significantly higher LSI-A scores (p = 0.002). However, no significant differences were observed in the mean LSI-A scores with respect to smoking, alcohol use or fall history (p > 0.05) (Table 2).

**Table 2:** Mean LSI-A scores according to health-related behaviors, fall history and regular leisure time activity

	**n (%)**	**LSI-A score Mean (SD)**	**p-value**
**Regular physical activity**^**1**^			**t = -2.59**
Yes	36(27.1)	22.47(5.70)	**p = 0.01**
No	97(72.9)	19.51(5.87)	
**Smoking **^**2**^			F = 1.80
Current smoker	61(45.9)	20.59(6.02)	P = 0.168
Ex-smoker	25(18.8)	21.84(6.44)	
Non-smoker	47(35.3)	19.14(5.48)	
**Alcohol use**^**2**^			F = 0.92
Abstainer	104(78.2)	20.24 (6.04)	P = 0.431
Infrequent drinker	14(10.5)	22.07(4.93)	
Moderate drinker	10(7.5)	20.30(7.08)	
Heavy drinker	5(3.8)	17.00(3.87)	
**Regular leisure time activity **^**2**^			**t = 3.22**
Yes	34 (25.6)	23.05(6.29)	**p = 0.002**
No	99(74.4)	19.37(5.56)	
**Fall history during the last year (at least once)**^**2**^			t = -1.42
Yes	29(21.8)	18.93(5.44)	p = 0.58
No	104(78.2)	20.70(6.06)	

^1^Unpaired t test

^2 ^ANOVA (one-way)

The multiple linear regression analysis results showed that regular physical and leisure-time activities were significantly related to LSI-A scores (p = 0.02, p = 0.005, respectively; R ^2 ^= 0.112) (Table 3).

**Table 3:** Predictors of life satisfaction index scores by multiple linear regression analysis

Predictors	Standardized Coefficients (β)	Significance (p)
Income	-0.028	0.738
Regular physical activity^a^	0.200	0.020
Regular leisure time activity^b^	0.239	0.005

R = 0.334; R^2 ^= 0.112; Adjusted R^2 ^= 0.091

a = 1: performs physical activity;

0: does not perform physical activity.

b = 1: participates in regular leisure time activity;

0: does not participate in regular leisure time activity;

## Discussion

Life satisfaction among the elderly has become an important issue in geriatric care [12-14]. The prevailing literature shows that it is affected by various physical, emotional, social and mental conditions [11,35]. Iwatsubo and colleagues [36] reported a significant relationship between life satisfaction and physical disabilities, leisure-time activities, marital and mental health status and family relationships amongst retired people in France. The results of our study also indicate a significant relationship between life satisfaction and involvement in regular physical and leisure-time activities. We also observed significantly lower life satisfaction scores in those with low incomes than in those with moderate and high incomes. The overall mean LSI-A score of our study group was 20.3 ± 5.9, which was lower than that (24.2 ± 4.4) reported by Iwatsubo et al. [36]. It can be argued that the differing results from different countries may be linked to differences in socio-cultural circumstances and demographic profiles. McConatha et al. reported that studies regarding life satisfaction of Turkish elderly population are few in number [4]. Imamoglu and Imamoglu [37] also compared Turkish respondents with those of a comparable Swedish sample. The findings of their cross-cultural study reported that, although Turks had more social contact with their relatives and neighbors, they had more negative attitudes about aging, felt lonelier, and had lower life satisfaction than Swedes. These authors also speculated that a decrease in satisfaction with social contacts may result in urban contexts, lowering life satisfaction for Turkish elderly.

However, in Turkey, Subasi and Hayran [38] found higher LSI-A scores (mean: 25.2 ± 5.5) than ours, which may be attributable to socio-cultural differences, vis-à-vis higher education and income levels, of the participants in the two studies. It may therefore be concluded that socioeconomic and educational variables are important factors in measuring the life satisfaction of the elderly both nationally [38] and internationally [39,40].

The mean LSI-A score for the physically active individuals in our study group was significantly higher than for those who led comparatively sedentary life styles (p = 0.01), a finding that is consistent with several other reports [35,41,42]. A previous study showed that daily walks, callisthenic exercises and/or Tai-Chi amongst nursing home residents were associated with higher life satisfaction scores [43]. Similarly, Subasi and Hayran [38] reported higher life satisfaction scores for elderly people who take part in regular recreational activities. The multivariate analysis of our data showed that leisure-time activities and regular physical activities were significant predictors of LSI-A scores. Thus, these results suggest the importance of encouraging the residents of nursing homes to take part in regular physical and/or leisure-time activities.

Regular physical activity as an important component of successful aging produces significant health benefits: it improves the health and functional status of the elderly [17] and also decreases the number of falls [20,21]. Approximately 30% of over-65s suffer at least one fall a year [20-23]. Elderly residents of nursing homes face a higher rate of falls because of nursing home settings and impaired functional capacities [21,23]. Nonetheless, in our study, the annual rate of falls (21.8%) was lower than others have reported. We believe that the low fall rate of our study group may be related to the restriction of our study to those who were ADL-independentand had sufficient cognitive functions.

Although life expectancy at birth is higher for females (74.4 years) than males (69.2 years) in Turkey, and elderly females slightly outnumber males in the population [3,44], more males thanfemales were included in our study group. In Turkey, approximately 65% of elderly living in the institutions is aged between 60 and 79 years [4]. It has been pointed out that Turks are generally more unfavorable toward institutional living [4,37]. It has also been implied that there are significant cultural differences between Turkey and western countries, which extend to attitudes toward aging and older adults. Turkey can be said to be a more collectivist culture. The studies indicated that older adults still hold considerable prestige and are valued and respected in Turkey [4]. Thus, only a relatively small percentage of the elderly population prefers to live in institutions in Turkey compared to industrialized western countries [44].

Generally speaking, most Turkish elderly either live alone in their homes in close proximity to their children or together with their married children [3]. However, Turkish males have more social network interactions than Turkish females. Relative to males, females are more often in relationships with their children, parents, and neighbors [44]. Compared to males, elderly females in particular are more frequently invited to live with their children and are rarely left to live alone [3]. Therefore, 60% of elderly people living in institutions in Turkey are male [5]. This might explain the higher proportion of males in our study, indicating a different aspect of the cultural and social structure of the country.

Acculturation is increasingly becoming an integral function of nurses and administrators in care organizations for elderly [45]. Therefore, the trends in Turkey in searching for alternative health care services for elderly people are becoming an important issue, similar to western countries.

Like any study, ours has some limitations. The first is the size and selection of the sample. Since our sample was selected from amongst the residents of a publicly owned institute in Istanbul, generalizing our findings to the entire Turkish elderly population may be somewhat tenuous. Also, our findings should be carefully interpreted in view of the slightly lower internal consistency (Cronbach's Alpha = 0.66) of the LSI-A in our study group.

## Conclusion

Our cross-sectional study data suggest that life satisfaction among elderly residents of institutions might be positively affected by participation in regular leisure and physical activities. We believe that further studies, designed longitudinally, may provide better information on the effectiveness and the sustainability of these activities on the life satisfaction level of elderly nursing home residents.

## Competing interests

The author(s) declare that they have no competing interests.

## Authors' contributions

SI conceived and supervised the study. SMA, SI and FS collected data. OH performed the statistical analysis. OH provided a critical review of the manuscript and statistical analysis. SI, FS and OH wrote and prepared the manuscript. All authors read and approved the final manuscript.

## Pre-publication history

The pre-publication history for this paper can be accessed here:


